# Improving metabolic parameters of antipsychotic child treatment (IMPACT) study: rationale, design, and methods

**DOI:** 10.1186/1753-2000-7-31

**Published:** 2013-08-15

**Authors:** Gloria M Reeves, Courtney Keeton, Christoph U Correll, Jacqueline L Johnson, Robert M Hamer, Linmarie Sikich, Lindsey Hazzard, Cheryl Alderman, Abigail Scheer, Micah Mabe, Sandeep Kapoor, Eva Sheridan, Irmgard Borner, Kristin Bussell, Sara Pirmohamed, Terrence C Bethea, Raja Chekuri, Rhoda Gottfried, Shauna P Reinblatt, Erin Santana, Mark A Riddle

**Affiliations:** 1 Department of Psychiatry and Behavioral Sciences, Division of Child and Adolescent Psychiatry, School of Medicine, Johns Hopkins University, Bloomberg Children’s Tower 12th Floor, 1800 Orleans Street, Baltimore, MD 21287, USA; 2 Division of Child and Adolescent Psychiatry, School of Medicine, University of Maryland, 701 W Pratt Street, Baltimore, MD 21201, USA; 3 Department of Psychiatry, University of North Carolina, 101 Manning Drive CB #7167, Chapel Hill, NC 27599, USA; 4 Division of Psychiatric Research, North Shore – Long Island Jewish Health System, Zucker Hillside Hospital, 75-59 263rd St, Glen Oaks, NY 11004, USA

## Abstract

**Background:**

Youth with serious mental illness may experience improved psychiatric stability with second generation antipsychotic (SGA) medication treatment, but unfortunately may also experience unhealthy weight gain adverse events. Research on weight loss strategies for youth who require ongoing antipsychotic treatment is quite limited. The purpose of this paper is to present the design, methods, and rationale of the Improving Metabolic Parameters in Antipsychotic Child Treatment (IMPACT) study, a federally funded, randomized trial comparing two pharmacologic strategies against a control condition to manage SGA-related weight gain.

**Methods:**

The design and methodology considerations of the IMPACT trial are described and embedded in a description of health risks associated with antipsychotic-related weight gain and the limitations of currently available research.

**Results:**

The IMPACT study is a 4-site, six month, randomized, open-label, clinical trial of overweight/obese youth ages 8–19 years with pediatric schizophrenia-spectrum and bipolar-spectrum disorders, psychotic or non-psychotic major depressive disorder, or irritability associated with autistic disorder. Youth who have experienced clinically significant weight gain during antipsychotic treatment in the past 3 years are randomized to either (1) switch antipsychotic plus healthy lifestyle education (HLE); (2) add metformin plus HLE; or (3) HLE with no medication change. The primary aim is to compare weight change (body mass index z-scores) for each pharmacologic intervention with the control condition. Key secondary assessments include percentage body fat, insulin resistance, lipid profile, psychiatric symptom stability (monitored independently by the pharmacotherapist and a blinded evaluator), and all-cause and specific cause discontinuation. This study is ongoing, and the targeted sample size is 132 youth.

**Conclusion:**

Antipsychotic-related weight gain is an important public health issue for youth requiring ongoing antipsychotic treatment to maintain psychiatric stability. The IMPACT study provides a model for pediatric research on adverse event management using state-of-the art methods. The results of this study will provide needed data on risks and benefits of two pharmacologic interventions that are already being used in pediatric clinical settings but that have not yet been compared directly in randomized trials.

**Trial registration:**

Clinical Trials.gov NCT00806234

## Introduction

In recent years, there has been growing concern about potential harm caused by second-generation antipsychotic (SGA) treatment of children and adolescents. SGA metabolic adverse effects include weight gain, dyslipidemia, increased blood pressure, and hyperglycemia/new onset diabetes [1-4]. Children and adolescents, especially those who are antipsychotic-naïve, are at greater risk for unhealthy weight gain adverse events than adults [3,5,6], and they often gain over 7% baseline weight within the first few months of treatment [1,7,8]. Providers may utilize pharmacologic (e.g. switch to a different SGA) and/or non-pharmacologic (e.g. diet/nutrition counseling) strategies to reduce adverse metabolic effects, but these interventions have not been systematically studied in pediatric patients [9]. Alternatively, prescribers may consider use of first-generation or “typical” antipsychotic medications. Some typical antipsychotic medications are associated with lower risk of obesity related adverse events compared to several SGA’s [3]. However, these older medications are associated with a higher risk of acute extrapyramidal adverse events and involuntary abnormal movements [6]. Of particular concern is the increased risk of tardive dyskinesia, a potentially irreversible movement disorder that typically manifests after long-term treatment.

This article reports on the rationale, design, and methods of a federally funded, multi-site clinical trial focused on management of metabolic adverse effects among youth who require ongoing antipsychotic medication treatment to maintain psychiatric stability. The Improving Metabolic Parameters in Antipsychotic Child Treatment (IMPACT) study is an open-label, 6-month trial, in which overweight and obese youth with SGA-induced weight gain are randomized to treatment with either (1) switch antipsychotic medication to one that is lower risk for metabolic side effects + healthy lifestyle education (HLE); (2) add metformin + HLE; or (3) HLE alone (control condition). This study began enrollment in 2008 and is being conducted at Johns Hopkins University, University of Maryland, University of North Carolina, and The Zucker Hillside Hospital.

### Rationale for study

SGA medications are often used to treat chronic and serious mental illness in adults and youth. Current FDA approved pediatric indications include irritability associated with autism, acute psychosis in schizophrenia, and mixed and manic episodes in bipolar disorder [10]. SGAs may cause significant adverse effects, but ongoing treatment may be required to achieve or maintain psychiatric stability. Three general strategies to manage SGA-induced weight gain include (1) switch antipsychotic medication to another antipsychotic with lower metabolic risk [11]; (2) add a pharmacologic weight loss agent [12]; and/or (3) add a non-pharmacologic weight loss intervention [13]. While these strategies are currently utilized in clinical settings, there is very limited pediatric data to guide clinicians on the risks and benefits of these strategies [9]. Randomized studies testing an antipsychotic switch strategy have only been conducted in adults, showing some reductions in weight and metabolic parameters [11,14]. Non-pharmacologic weight loss interventions for adult antipsychotic-related weight gain have had some limited success, yet no such studies exist in pediatric patients [9,13]. Further, no pediatric RCT’s have compared strategies to manage antipsychotic induced weight gain. In contrast, two adult studies have been conducted that directly compared different weight loss strategies in antipsychotic treated patients. Wu et al. [15] compared metformin, healthy lifestyle, and the combination of metformin plus healthy lifestyle, and Stroup et al. [16] compared healthy lifestyle intervention versus healthy lifestyle plus switch to a lower cardiometabolic risk antipsychotic. To date, no three-arm study of weight loss options for antipsychotic treated patients who experienced relevant weight gain exists, and no study has directly compared addition of a weight loss medication with a switch to a lower risk antipsychotic and healthy lifestyle instructions.

Unhealthy weight gain caused by pediatric SGA treatment is a major public health concern because childhood obesity is a risk factor for cardiovascular disease and type 2 diabetes. The prospective Harvard Growth Study reported that being overweight in adolescence is a more powerful predictor of morbidity from coronary heart disease than being overweight as an adult [17]. A large population cohort epidemiologic study reported that the risk for any adult coronary heart disease was positively associated with body mass index (BMI) at age 7–13 for boys and 11–13 for girls, and the risk increased across the entire BMI distribution [18]. Obesity is also the most important risk factor for development of pediatric type 2 diabetes [19]. Sinha et al. [20] reported a high prevalence of glucose intolerance (25% of children; 21% of adolescents) and 4% with silent type 2 diabetes among a sample of obese youth. Although youth treated with SGAs seldom develop diabetes because of their large insulin reserve, they often develop insulin resistance and dyslipidemia, which markedly enhance the risk for long-term morbidity and mortality. Unfortunately, childhood obesity often persists into adulthood [21-23]. For example, in the Bogalusa Heart study, 77% of obese children were obese as young adults [24].

IMPACT is the first pediatric study to systematically examine more than one active treatment for SGA induced weight gain using a longitudinal design and state-of-the-art methodology. The results of this study will guide future clinical interventions to provide the safest treatment for youth prescribed SGAs, namely those with early onset schizophrenia spectrum, pediatric bipolar disorder, severe mood dysregulation, psychotic or non-psychotic major depressive disorder, or irritability associated with autistic disorder.

### Rationale for intervention arms

#### Switch antipsychotic medication

Several expert consensus statements [3,25,26] recommend switching of the antipsychotic to a lower metabolic risk agent as an option to manage antipsychotic induced weight gain. This option has the advantage of removing the offending agent and providing ongoing treatment at therapeutic doses (as opposed to the strategy of lowering the dose). However, there is a potential risk for psychiatric de-stabilization, even with a careful cross-over titration. A 2010 Cochrane review of four randomized controlled trials (RCTs) of antipsychotic switch versus continued treatment in adults concluded that switching to a lower risk agent may be an effective strategy to manage metabolic side effects, resulting in weight loss and improved fasting blood glucose [11]. Of note, there was no difference in adverse events (including or excluding metabolic adverse events) in the switch versus continuation antipsychotic groups in these preliminary studies.

Selection of an appropriate switch agent for the IMPACT trial was challenging because of rapid changes in available SGAs. Initially, aripiprazole was selected as the switch agent, based on promising data from both adult and pediatric psychiatry studies that this agent had a lower risk of adverse metabolic events [27,28]. This information lead to significant increases in off-label prescribing, even before pediatric data from controlled efficacy and safety studies were available. Aripiprazole quickly became a first line prescribed antipsychotic medication. Unfortunately, evidence emerged that, at least among antipsychotic naïve patients, aripiprazole can also be associated with significant weight gain. For example, in a non-randomized inception cohort study, antipsychotic-naïve youth prescribed aripiprazole experienced a mean weight gain of 4.4 kg at 12 weeks, which was less than the weight gain of 8.4 kg on olanzapine, 6.1 kg on quetiapine and 5.3 kg on risperidone, but significantly greater than the 0.19 kg weight gain experienced by non-antipsychotic treated youth [1]. Thus, we required an appropriate alternative switch medication for youth who had either inadequate prior response to aripiprazole and/or weight gain on this medication.

Molindone was selected as an alternative switch agent. This choice was based largely on the results of a double blind RCT for youth with schizophrenia or schizoaffective disorder in which weight gain at 8 weeks was greatest for olanzapine (6.1 kg), followed by risperidone (3.6 kg), and molindone (0.3 kg) [29]. Shortly after being added to the IMPACT study switch arm, however, molindone was removed from the market by the manufacturer for non-safety reasons. Only one participant received this medication, and the parent elected to discontinue treatment since the child could not stay on the medication after the study was completed.

Perphenazine was selected to replace molindone as the second antipsychotic switch medication. There are no modern, placebo-controlled, randomized trials of perphenazine in youth. The one published study [30] has too many methodological problems to be interpretable. Perphenazine was selected based on the CATIE study, an adult schizophrenia trial that compared perphenzine to olanzapine, quetiapine, and risperidone treatment [31]. Perphenazine had a better profile in terms of drug-induced weight gain and metabolic changes compared to the SGA’s, but had a higher discontinuation rate for extrapyramidal side effects. To address the potential risk of EPS, we provided prophylactic treatment with benztropine for youth prescribed > 8 mg perphenazine. EPS symptoms were monitored at each visit (refer to Table 1 for assessment schedule). For all participants, the protocol allows for treatment of emergent EPS with benztropine or trihexyphenidyl and treatment of akathisia with propranolol, lorazepam, or clonazepam.

**Table 1:** Schedule of major study assessments

**Assessment**	**Screening**	**0**	**1**	**2**	**4**	**6**	**8**	**12**	**16**	**20**	**24**
**Diagnostic evaluation**											
Kiddie schedule of affective disorders – present/lifetime	X										
Aberrant symptom checklist	X										
Wechsler abbreviated scale of intelligence	X										
Family history	X										
Medical history	X										
Urine drug screen	X										
**Psychiatric stability (blinded rating)**											
Clinical global impression – severity and improvement scales		X						X			X
Brief psychiatric rating scale for children		X						X			X
**Major metabolic assessments**											
Weight, height (body mass index z-score)	X	X	X	X	X	X	X	X	X	X	X
Waist circumference	X	X						X			X
Fasting glucose, insulin	X	X						X			X
Optional OGTT		X						X			X
HDL, LDL, triglycerides		X						X			X
DEXA		X						X			X
Metabolic syndrome		X						X			X
**Medication tracking**											
Pill count (study drug) and self-report			X	X	X	X	X	X	X	X	X
Drug level (SGA, metformin)*		X						X			X
Review of co-prescribed and OTC medication											
**Adverse event/safety monitoring**											
Barnes Akathisia scale	X	X	X	X	X	X	X	X	X	X	X
Simpson Angus extrapyramidal symptoms scale	X	X	X	X	X	X	X	X	X	X	X
Abnormal involuntary movement scale		X					X				X
ECG	X										X
Urine pregnancy (females)	X	X			X		X	X	X	X	X
**HLE review**		X	X	X	X	X	X	X	X	X	X

Ziprasidone is associated with lower metabolic adverse effects, but it was not chosen as a switch agent because it has a relatively greater risk for QTc prolongation compared to other antipsychotic medication options [3]. Ziprasidone treatment would require greater cost and time for ECG monitoring to address potential increased risk of arrhythmias. A recent study indicates that this risk is likely not clinically relevant [32], however, ECG monitoring continues to be done as part of standard of care for ziprasidone treatment.

*Antipsychotic switch to a lower risk agent*, either aripiprazole or perphenazine, represents a single weight loss strategy in IMPACT. The analysis will not distinguish between aripiprazole and perphenazine or examine any differences between them, except descriptively, because the selection of the switch medication is non-random, and based on prior treatment history.

#### Add metformin

The selection of a pharmacologic weight loss agent was based on pediatric safety/tolerability, feasibility, and efficacy data specifically for SGA-induced weight gain. Four medications were considered: sibutramine, orlistat, topiramate, and metformin. Sibutramine and orlistat received FDA approval for treatment of obese adolescents (orlistat for youth ≥ 12 years old; sibutramine for youth ≥16) [33]. Sibutramine is a norepinephrine and serotonin re-uptake inhibitor that promotes weight loss via appetite suppression and increases in resting energy expenditure [34]. Sibutramine was not selected for the IMPACT study because of potential for adverse CNS effects, and it subsequently was withdrawn from the US market in 2010 because of increased risk of heart attack and stroke in adults [35]. Orlistat is an enteric lipase inhibitor [36]. It was not selected because of concerns that adherence may be hindered by adverse effects, including fecal incontinence [37], as well as by the complexity of the medication regimen, i.e., it is recommended to administer a multi-vitamin at least 2 hours apart from orlistat and to use this medication up to three times a day with meals [38].

Topiramate and metformin have been studied specifically for antipsychotic-induced weight gain in adults. Topiramate is an antiepileptic agent that has FDA pediatric approval down to age 2 for seizure treatment [39]. It is also used for off-label treatment of mood disorders, with the frequently reported side effect of decreased appetite/weight loss. A review by Ellinger et al. [40] reported topiramate was superior to placebo in three adult RCT’s for either weight loss or BMI outcome (in this study the topiramate goup had stabilization of BMI rather than significant reduction in BMI compared to BMI increase in placebo group). However, concerns about topirimate use include potential drug interaction (25% decrease in risperidone concentrations), troublesome neurologic adverse events (paresthesias reported more commonly in the topiramate treated groups in two of the trials), and the 2008 FDA warning about increased risk of suicidal behavior or ideation among patients taking antiepileptic medications [40].

Metformin was selected as the weight loss agent for the IMPACT study. Metformin decreases hepatic gluconeogenesis and improves insulin sensitivity in the liver and muscle [41]. Metformin is FDA approved for the treatment of type 2 diabetes in youth ≥10 years old [42]. The potential side effect of hypoglycemia secondary to metformin is rare because metformin does not stimulate insulin production, and there have been no published pediatric cases of lactic acidosis or increase in serum lactic acid associated with therapeutic doses [43]. A recent review [44] reported on 13 published trials, including 2 pediatric and 6 adult RCTs, of metformin to manage or prevent antipsychotic induced weight gain, which the authors summarized as being associated with modest weight loss demonstrated in mostly short term trials. Another systematic review and meta-analysis on the effect of metfomin treatment on antipsychotic-induced weight gain [45] reported weight change compared with placebo as −4.8% in adults and −4.1% in children, although this analysis only included two pediatric studies. One study was a 12-week RCT in which youth treated with risperidone were augmented with metformin or placebo, and both groups showed significant changes in weight and BMI, however the study included only 32 youth [46]. The other pediatric study was a 16-week RCT examining metformin co-treatment for 10–17 year old youth (n = 38) prescribed olanzapine, risperidone, or quetiapine [47]. There were significant differences in weight change between the metformin and placebo treated groups, but the metformin group did not experience significant weight loss; i.e., the placebo treated group continued to gain weight while the metformin group maintained a stable weight.

#### Control condition

All participants receive healthy lifestyle education. Because of the clear health risks of pediatric overweight and obesity, it would be unethical to withhold non-pharmacologic interventions for six months during the course of the study. When the IMPACT study was initially proposed, grant reviewers raised concern about funding an intensive psychosocial intervention (e.g. diet and activity interventions at a weight management center) because there was no substantial evidence base that this type of intervention would be effective for mentally ill youth receiving antipsychotic medication. Another concern was the potential high burden of frequent and long study visits for patients receiving lifestyle intervention plus pharmacologic intervention, and the possible increased parent–child conflict with a highly restrictive dietary or physical activity intervention.

The basic healthy lifestyle education employed in IMPACT follows stage 1 of the American Medical Association pediatric weight loss guidelines (http://www.ama-assn.org//ama1/pub/upload/mm/433/ped_obesity_recs.pdf). This educational program can be administered by a prescriber or non-clinician and can be easily implemented in a mental health outpatient setting, even with time constraints. The duration of the lifestyle education is consistent with guidelines from the 2009 Schizophrenia Patient Outcomes Research Team report [48] that indicates such an intervention should occur for at least 3 months. To our knowledge, the AMA pediatric weight loss guidelines have not been studied in a pediatric weight loss trial for youth with mental illness. Stage 1 and stage 2 guidelines are provided by the AMA.

Stage 1 guidelines focus on education about healthy diet (e.g. eat breakfast daily) and activity habits and can be implemented by a non-clinician. HLE is advanced to Stage 2 guidelines if he/she gains 7% of baseline weight. Stage 2 guidelines address the following:

1. Eating habits: Increased structure of daily meals and snacks.

2. Food consumption: Develop a balanced diet with low amounts of energy dense foods,

3. Physical activity: Supervised active play 60 minutes per day, limit sedentary activity involving electronic devices, such as television or computer to <1 hour per day

4. Monitoring of food consumption and activity by parent or child and provider.

### Specific aims and design

**Figure 1:**
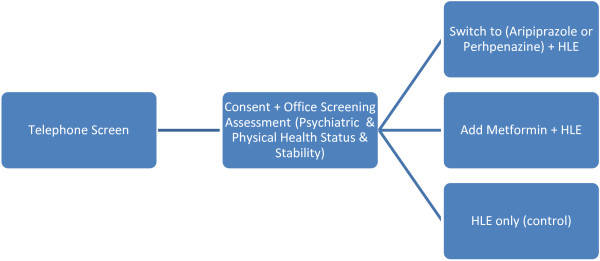
**Screening to randomization flow chart.** Note: HLE = Healthy Lifestyle Education.

IMPACT is a 6-month, multi-site study involving an initial screening phase to assess eligibility followed by 24 weeks of randomly assigned open-label treatment and monitoring (Figure 1). This study was approved by the Institutional Review Boards at the four sites.

#### Specific aims and hypotheses

*The primary aim* is to determine if each of the pharmacologic interventions (switch antipsychotic; add metformin) results in greater weight loss, as measured by BMI z-score change over 6 months, compared to the control condition (healthy lifestyle education). As part of secondary analyses, weight loss will also be examined as 6-month change in BMI percentile, absolute body weight, percent body weight, and total body fat assessed with Dual-Energy X-Ray Absorptiometry (DEXA). We hypothesize that mean change in BMI z-score and body fat mass, as a percent of baseline body fat mass, will be reduced over six months in each of the treatment groups compared to the control group.

*The first secondary aim* is to determine if the two medication strategies result in greater improvement in metabolic measures, including insulin resistance, triglycerides, LDL cholesterol, and point prevalence of metabolic syndrome (using adolescent criteria [49]), compared to the control condition. We hypothesize that these metabolic parameters will improve in each of the treatment groups compared to the control group.

*The second secondary aim* is to establish whether the proportion of subjects discontinuing treatment is lower in each pharmacologic group compared to the control group. Exploratory analyses will examine the proportion of subjects in each group who discontinue assigned treatment (HLE, or HLE plus metformin or switch antipsychotic) because of 1) psychiatric symptoms and 2) weight or metabolic reasons. We hypothesize that fewer participants will discontinue treatment in each of the treatment groups compared to the control group since we anticipate that lifestyle education may not be intensive enough to promote weight loss while the child continues on antipsychotic treatment. We recognize that antipsychotic switch assignment has a risk of de-stabilizing psychiatric symptoms, however our protocol allows for a gradual cross-over titration and flexible dosing to minimize adverse events during the medication switch.

The most important *exploratory analyses* will compare the two pharmacologic intervention groups with respect to each of the primary and secondary aims. We hypothesize that the metformin group will have greater reduction in insulin resistance compared to the switch antipsychotic group, but that the switch antipsychotic group will have greater reduction in triglycerides than the metformin group.

We hypothesize that the metformin group will have a greater reduction in insulin resistance compared to the switch antipsychotic group because metformin is a diabetes medication that is specifically used to improve insulin sensitivity. This medication is not known, however, to have specific effects on lipid profile. Thus, we anticipate that removing the offending agent (i.e. the antipsychotic causing the weight gain) in the switch arm will be more effective in reducing abnormal triglycerides than metformin.

#### Study design

### Participant sample

Eligibility criteria identify youth aged 8–19 years who have benefited enough from antipsychotic treatment to justify ongoing treatment (i.e., achieved psychiatric stability – for definition, see Table 2), but who are also at *greatest risk for harm from SGA related weight gain*. Only youth who: 1) meet CDC criteria for being “overweight” (i.e., 85^th^- < 95^th^ BMI percentile) or “obese” (i.e., >/=95^th^ BMI percentile), and 2) have been identified by both their caregiver and clinician as having experienced “substantial weight gain” during SGA treatment in the past 3 years, are eligible. These pediatric patients have a compelling need for more intensive side effect monitoring. Also, their current vulnerability to obesity-related health problems balances the risk of being randomized to an intervention that is not yet widely used in child psychiatric clinical settings (i.e., addition of metformin). Full inclusion and exclusion criteria are presented in Tables 2 and 3.

**Table 2:** Inclusion criteria and rationale

**Criterion**	**Rationale**
Ages 8 to 19 years	Even young children are frequently treated with SGAs and experience severe weight gain. The FDA has approved metformin for use in type 2 diabetes in children ≥ 10 years, it appears 8 year olds could safely use metformin based on available toxicology literature (Spiller et al. 2000; Benavides et al. 2005), use in previous clinical trials (Ibanez et al. 2004; Lutjens and Smit 1977), and advice from our pediatric endocrinology consultant.
Primary DSM-IV diagnosis of Early Onset Schizophrenia Spectrum disoder, Bipolar Spectrum disorder, Major Depression with psychotic features, Major Depressive Disorder (only for participants aged 18–19 years), or Autism with irritability	Most diagnoses or symptom clusters for which a SGA is prescribed in clinical practice are included to enhance ecological validity.
Clinically stable on current treatment regimen for ≥ 30 days	Stability is required to reduce risk of psychiatric decompensation
Stable dose of current SGA and psychotropic co-medications for ≥ 30 days	
BMI ≥ 85th percentile for age and gender (i.e., at least “at risk for overweight”)	Youth at *greatest risk for harm from SGA weight gain* need intensive side effect monitoring. Current vulnerability to overweight/obesity-related health problems balances the risk of being randomized to an intervention that is not yet widely used in child psychiatry clinical settings (i.e. addition of metformin).
Substantial weight gain over the previous 3 years, while taking a SGA (aripiprazole, asenapine, iloperidone, lurasidone, olanzapine, paliperidone, quetiapine, risperidone, or ziprasidone)	All SGAs other than clozapine were included because all have been associated with substantial weight gain Clozapine was not included because of unique benefits and it is generally reserved for youth with severe illness that has not been adequately controlled with other antipsychotics.
Sexually active girls must agree to use two effective form*s* of birth control (i.e., hormonal *o*r spermicidal *and* barrier) or be abstinent	Risk of study agents to unborn babies.
Participant has a primary caretaker (defined as parent(s), close relative functioning *in loco parentis*, legal guardian, or foster parent) who has known the child well for at least 6 months before study entry	Legal authority to make medical decisions including participation in research study. Ability to accurately report on past and current functioning.
Primary caretaker is able to participate in study appointments as is clinically indicated.	Most participants are minor children.
Ability of child to participate in all aspects of the protocol per investigator clinical judgment.	Child must demonstrate awareness of study procedures and assent to participate.
After considering all aspects of study participation, including random assignment, guardian and the child must agree (legally consent and assent) to participation	Study participation is voluntary but requires consent.

**Table 3:** Exclusion criteria

**Eligibility screening information**	**Exclusion criteria**
Current medication regimen*	1. Any medication that would significantly alter glucose, insulin, or lipid levels.
	2. Treatment with >1 antipsychotic medication.
	3. Treatment with >3 total psychiatric medications (exception: 4 total permitted if 2 are ADHD drugs)
Screening labs**	4. Fasting glucose >125 mg/dL
	5. Positive urine toxicology screen
	6. Serum creatinine ≥ 1.3 mg/dL
	7. Independent pediatric consultation is required if glucose 100-125 mg/dL; triglycerides >300 mg/dL, total cholesterol >300 mg/dL, ALT>174 or 3X upper limit normal to determine if study participation is appropriate
Somatic health conditions***	8. Any major neurological or medical illness that affects weight, requires a prohibited systemic medication, or prohibits physical activity/use of AMA weight loss guidelines.
**Psychiatric diagnosis**	9. Substance dependence disorder (except tobacco dependence) in the past month
	10. Current or lifetime diagnosis of anorexia nervosa or bulimia nervosa
	11. IQ <55
**Medication history**	12. Known hypersensitivity to aripiprazole, perphenazine, or metformin
	13. Prior trials of aripiprazole and perphenzine that were >2 weeks and stopped because of efficacy or tolerability concerns
	14. Psychiatric medication or dosage change within the past 30 days.
**Additional safety concerns**	15. Significant risk of dangerousness to self or others
	16. Ongoing or previously undisclosed child abuse requiring new social service intervention.
**Caregiver considerations**	17. Caregiver unable to participate in assessments or has not known child at least 6 months
	18. Caregiver or child have language issue that makes them unable to complete assessments
**Female participants**	19. Pregnant, nursing, or sexually active and unwilling to comply with double method contraceptive

*Antipsychotic medication, stimulant medication, and divalproex sodium are permitted. Prohibited medications include, but are not limited to: insulin, systemic steroids, topiramate, sibutramine, orlistat, metformin, amantadine, vitamin E (other than in standard multivitamins), statins, and anti-hypertensive medication. Exception: orlistat and amantadine are permitted if taken for >1 year without weight loss.

**Laboratory tests are repeated for confirmation if they are abnormal.

***Medical conditions that lead to exclusion include, but are not limited to, diabetes, unstable thyroid disease, chronic renal failure.

We have operationalized “substantial weight gain” to be approximately equivalent to at least 10% of baseline weight. We allow some flexibility in this determination because of challenges in obtaining precise and accurate clinical weight records (e.g. challenges weighing a child who may have developmental delay and hyperactivity behavioral problems; child may have been weighed on different scales using different procedures over the course of antipsychotic treatment, etc.). When a child’s weight is measured at the screening visit and over the course of the study, we have systematic, study-wide procedures (e.g. same type of scale used; height measured three times to calculate sex- and age-adjusted BMI percentiles; removal of shoes and added layers of clothing like a jacket are removed prior to measurement) to improve the accuracy of weight and ability to compare weights across sites.

Of note, two conditions are permitted as part of the eligibility criteria to maintain generalizability of the findings to a typical child psychiatry clinical population. First, we allow for ongoing stable treatment with either a stimulant medication and/or valproate/valproic acid. Both of these medications are known to effect weight, but we did not exclude them because they are commonly prescribed to youth receiving antipsychotic medication. We monitor adherence to stimulants and valproate/valproic acid for youth receiving these medications at each study visit along with adherence of antipsychotic medication and study medication. We also restrict the total number of psychiatric medications permitted (3 medications total, including antipsychotic medication or 4 medications total, if 2 are prescribed for ADHD). Secondly, in addition to including diagnoses that either have a pediatric or adult approved indication, we include youth receiving off-label antipsychotic treatment for chronic mood dysregulation. Chronic mood dysregulation is operationalized using the Leibenluft [50] criteria for Severe Mood Dysregulation (SMD). Each case is reviewed by the PI steering committee to provide final approval for randomization based on the eligibility criteria and clinical status and stability over the screening period.

### Randomization

Eligible participants are randomly assigned to one of the three treatment conditions using a central, computer-generated randomization schedule developed and administered by the Data Center (DC). Randomizations is stratified for current SGA (risperidone, aripiprazole (the two largest expected groups) or “other antipsychotic”) and diagnosis (1. Early Onset Schizophrenia Spectrum or 2. Bipolar Disorder, Severe Mood Dysregulation, psychotic or non-psychotic depression, or irritability associated with autism). We decided to stratify randomization between the two major diagnostic groups, in order to account for potential differences in sensitivity to weight gain and in co-medications. In addition, we stratified by the two baseline SGAs that we expected to produce the largest groups, risperidone, aripiprazole and “other antipsychotic”, as the weight gain potential and metabolic liability of these antipsychotic groups differs, leading to potentially different magnitudes of change in response to the randomized treatment.

### Treatments

#### Healthy lifestyle education (all subjects)

All participants in the study receive the healthy lifestyle education following the stage 1 of the 2007 American Medical Association guidelines for treatment of pediatric obesity, which provides guidance on healthy eating habits and activities. Participants advance to stage 2 if they gain >7% of their baseline body weight during the conduct of the study to minimize human subject concerns. Stage 2 increases supervision of activity and food intake and also reduces sedentary activity associated with television/computer viewing time.

#### Switch antipsychotic

Youth randomized to this condition have a switch in their antipsychotic treatment to an antipsychotic medication, with a lower metabolic risk profile, i.e., aripiprazole or perphenazine. Antipsychotic switch occurs in a gradual plateau cross over design [51] with flexible dosing (Table 4 and Table 5). The first line switch agent is aripiprazole, the second switch agent is perphenazine. Youth receive aripiprazole if they have never been treated with this agent or if they had a prior inadequate trial without inefficacy or intolerability; otherwise, they are treated with perphenazine. Participants who take perphenazine are prescribed benztropine, 0.5 mg twice a day if they are titrated to a dose >8 mg. This dose of benztropine may be adjusted (increased or discontinued) after it is initiated based on prescriber clinical judgment.

**Table 4:** Recommended dose equivalency for switch condition

**Entry SGA**	**Switch to aripiprazole**	**Switch to perphenazine**
Aripiprazole	--	Dose X 1.33
Asenapine	Dose X 1.5	Dose X 2
Iloperidone	Dose X 1.25	Dose X 1.75
Lurasidone	Dose X 0.25	Dose X 0.35
Olanzapine	Dose X 1.5	Dose X 2
Paliperidone	Dose X 2.5	Dose X 3.33
Quetiapine	Dose X 0.1	Dose X 0.13
Risperidone	Dose X 4	Dose X 5
Ziprasidone	Dose X 0.125	Dose X 0.17

**Table 5:** Recommended open-label switch titration dosing

	**Entry SGA**	**Aripiprazole**	**Perphenazine**
Week 0	100%	2 mg	4 mg
Week 1	100%	5 mg	8 mg
Week 2	100%	10 mg	12 mg
Week 3	75%	15 mg	16 mg
Week 4	50%	20 mg	24 mg
Week 5	25%	25 mg	28 mg
Week 6 (through week 24)	0%	30 mg^*^	32 mg^*^

^*^This target dose varies based on dose equivalency calculation (Table 5)

(Target dose based on dose equivalency calculation).

#### Metformin treatment

Metformin is administered using a flexible dosing titration schedule over 4–6 weeks (see Table 6 for general guidelines on dosing schedule and target dose based on two weight groups). The dose is maintained or lowered based on side effects. Youth who experience significant side effects are offered the extended release formulation of metformin, which has a lower incidence of gastrointestinal side effects. Families are provided with informational handouts describing potential drug interactions and the risks of hypoglycemia and hyperglycemia. Metformin dosing reaches steady state target levels before key outcomes are assessed at Week 12.

**Table 6:** Recommended open-label metformin (plus multivitamin) AM/PM titration dosing

	** <50 kg**		** 50-70 kg**		** >70 kg**	
	** am /**	**pm**	** am /**	**pm**	** am /**	**pm**
Week 0	0 mg /	250 mg	0 mg /	250 mg	0 mg /	500 mg
Week 1	250 mg /	250 mg	250 mg /	250 mg	500 mg /	500 mg
Week 2	250 mg /	500 mg	250 mg /	500 mg	500 mg /	1000 mg
Week 3	250 mg /	500 mg	500 mg /	500 mg	500 mg /	1000 mg
Week 4	500 mg /	500 mg	500 mg /	1000 mg	1000 mg /	1000 mg
Week 5	500 mg /	500 mg	500 mg /	1000 mg	1000 mg /	1000 mg
Week 6 (through week 24)	500 mg /	500 mg	500 mg /	1000 mg	1000 mg /	1000 mg

#### Control condition

Participants randomized to the control condition receive healthy lifestyle education only, and no changes are made to their medication.

### Measures and assessment of outcomes

Assessment time points are indicated in Table 6. There are 10 in-person visits, at 0, 1, 2, 4, 6, 8 and 12, 16, 20 and 24 weeks. In addition, there are telephone contacts during the first 11 weeks when no regular in-person visit is scheduled, i.e., at weeks 3, 5, 7, 9, 10 and 11. The major metabolic assessments occur at baseline, 12 weeks, and 24 weeks.

The primary outcome measure is the BMI z-score change (from baseline to week 24), which requires measurement of height and weight. BMI z-scores adjust for age- and gender-appropriate developmental changes. Further, BMI z-scores are continuous measures, in contrast to BMI percentiles, which are capped at the upper and lower end.

DEXA scan is used to measure change in total fat mass. This measurement was selected instead of percent body fat for two reasons: 1) smaller changes in total body fat mass can be detected than in percent body fat, thus the precision is greater, and 2) weight loss is clinically significant even if it equally affects fat and fat-free tissues.

All metabolic laboratory values obtained at the main visits (baseline, week 12, week 24) are obtained after an overnight fast of at least 8 hours. Glucose, insulin, triglycerides, total cholesterol, high-density lipoprotein (HDL)- cholesterol, and low-density lipoprotein (LDL)-cholesterol are analyzed centrally at the Mid-Atlantic Nutrition and Obesity Research Center at the University of Maryland School of Medicine. An oral glucose tolerance test (OGTT), the more definitive test of insulin sensitivity, was not required for all patients based on our experience that some youth would have difficulty tolerating this lengthy procedure but it was offered as an optional test. Thus, all participants will have insulin sensitivity measured by a fasting blood draw, and a sub-group will also have insulin sensitivity measured by an OGTT. We will analyze the sub-group of participants who completed both measurements separately to (1) assess correlation between the fasting blood draw and OGTT insulin sensitivity measurements; and (2) to compare OGTT derived insulin sensitivity results for the three study arms.

### Medication adherence

For all patients, parent/patient self-report adherence data are collected regarding all psychotropic medications, including antipsychotics, stimulants, and depakote at each medication visit. In addition, for all patients, antipsychotic blood levels are collected at week 12 and 24. Finally, for participants receiving study medication (i.e., switch antipsychotic or metformin, information is also collected by pill count at all medication appointments and by drug levels of metformin at week 12 and 24.

### Statistical methods and analytic plan

The primary aim is to compare the relative efficacy of two medication strategies (S = switch to lower risk agent, M = metformin augmentation) versus a control condition (C = control, current SGA) in reducing BMI z-score. Analyses will be performed on a modified intent-to-treat population of patients with a baseline and at least one follow-up visit, using a mixed model approach to repeated measures (MMRM). Change in BMI z-score as well as in secondary body composition parameters (i.e., change in BMI percentile, weight and percent weight compared to baseline, fat mass, waist circumference) will be modeled as a function of treatment group, time point, and treatment-by-time point interaction, with current SGA and diagnosis as blocking factors. The original IMPACT data analysis plan treated the individual treatment contrasts of (a) switch to lower risk agent versus control condition and (b) metformin versus control condition as co-primary hypotheses, using a 0.025 (=0.05/2) significance level via Bonferroni correction for each test. We also used 90% power, which produced an estimate of 240 patients required. This initial analysis plan was reviewed and revised after the first year of recruitment because of concerns that this approach may be overly conservative and the sample size may be difficult to achieve given the challenges of recruiting for this study (e.g. difficulty recruiting individuals with serious mental illness who meet the clinical stability criteria). The decision was made, prior to any data analysis, to re-structure the hypothesis testing to a two stage procedure, which reduced the sample size requirements. We will first test for a treatment group main effect in the three treatment group model. If this test is significant, we will then perform two follow-up tests comparing each of the treatment groups to control. The test of treatment main effect as well as the two follow-up tests will be conducted at significance level 0.05, instead of 0.025. This testing procedure does not inflate the overall experiment-wise error rate, since if we reject the group null hypothesis, we know at least one of the group means differs from at least one of the others. The sample size (132 total, 44 per group) was chosen to allow at least 80% power for the follow-up pairwise treatment group comparisons on change in BMI z-scores. This final sample size does not adjust for attrition. With respect to change in BMI z-scores, we hypothesized values of: μ_C_ = +0.25, μ_M_ = −0.15, μ_S_ = −0.10 based on published [47] and pilot data. We assumed a standard deviation of 0.58. With respect to change in total fat mass as a percent of baseline fat mass, we hypothesized values of: μ_S_ = +1.0%, μ_M_ = −6.0%, and μ_C_ = +18%. . We assumed a standard deviation of 20%.

The secondary aim is to compare the two medication strategies to the control condition on changes in metabolic measures, including glucose, insulin, insulin resistance (measured as Quantitative Insulin Sensitivity Check Index; QUICKI and Homeostasis Model of Insulin Resistance; HOMA-IR [52,53]), triglycerides, total cholesterol, LDL-cholesterol, non-HDL cholesterol, HDL-cholesterol, glucose and metabolic syndrome. In addition, in subjects undergoing the optional 2-hour OGTT, changes in whole body insulin sensitivity index (ISI), which utilizes insulin and glucose levels obtained at 30-minute intervals the OGTT, will be assessed. ISI will be calculated with the Matsuda & DeFronzo [54] formula: ISI = 10,000 divided by the square root of (fasting plasma insulin X fasting plasma glucose X mean plasma glucose X mean plasma insulin).

Hypotheses are that insulin sensitivity will be increased in each of the treatment groups individually compared to the control group, and that lipid and glucose and insulin levels will be reduced. These hypotheses will be tested using the same MMRM analyses as the hypotheses for the primary aim using changes in insulin resistance, percent reduction in the lipid and glucose metabolism parameters, and the same two stage testing procedure to compare treatment groups. With regard to metabolic syndrome, we will compare the proportion of subjects with metabolic syndrome at week 24 in the treatment groups using logistic regression, with group as a fixed predictor, SGA, and diagnosis as blocking factors.

Finally, our third aim is to compare each of the treatment conditions individually with the control group with respect to proportion of subjects discontinuing treatment. This will use a logistic regression, with group as a fixed predictor, and SGA and diagnosis as blocking factors. We hypothesize that fewer subjects will discontinue treatment in the metformin group or in the lower risk agent group than in control group. We plan to also examine the proportion of subjects in each group who discontinue treatment because of 1) psychiatric symptoms and 2) weight or metabolic reasons, and to examine time to discontinuation.

### Design weaknesses and limitations

A limitation of the IMPACT study is that it is an open label trial. Double blinding of all treatments was not done because of the additional costs to purchase current SGA; costs to over-encapsulate eight different medications (six antipsychotics, metformin, and placebo); the complexity related to changing dose of three different blinded medications simultaneously; the subsequent likelihood of errors and non-compliance; and the burden to participants associated with taking more and larger capsules each day. We did not feel that double blinding was necessary because the likelihood that our primary outcome measure, BMI z-score change, would be affected by bias is extremely low. Only BPRS-C and CGI ratings are likely to be influenced by open treatment. To provide adequate protection against bias, single-blind, independent evaluation of psychiatric symptomatology were included for these measures.

Another weakness of the study is that the healthy lifestyle intervention used for the control condition offered only basic educational support. It is possible that a more intensive intervention, including a supervised exercise component and/or more intensive diet monitoring, may be more effective in promoting weight loss than pharmacologic interventions. However, we sought to test interventions that could be easily administered in a mental health setting and would not require additional nutritional or other specialist expertise.

Finally, another limitation is that the oral glucose tolerance test was offered as an optional assessment. All participants had measurement of insulin sensitivity (using fasting glucose and insulin), but the more sensitive measure of insulin sensitivity (OGTT) was not required because of the concerns about burden of testing on participant. The study included youth as young as age 8, and the two hour OGTT required children to fast until the metabolic blood work was completed. The measurements selected (QUICKI and HOMA-IR) to measure insulin sensitivity in all participants were done based on consultation with our study-wide and site endocrinology experts.

### Safety & ethical considerations

Important safety and ethical concerns addressed in the study design focus on safeguards to ensure that (1) the risk to benefit ratio for the individual patient is appropriate to continue antipsychotic treatment for six months; (2) stopping criteria are established for youth who experience psychiatric de-stabilization; and (3) increased lifestyle interventions are permitted for those who continue to gain weight.

To insure that an individual patient is appropriate for continued antipsychotic treatment, each case must be approved by a PI steering committee prior to enrollment. The diagnostic evaluation, clinical laboratory studies, and the patient’s current functioning are reviewed.

For patients who continue to gain weight during the trial, additional healthy lifestyle intervention is provided. For youth who gain >5% baseline weight, an additional session is scheduled to review stage 1 AMA guidelines. For youth who gain >7% baseline weight, the stage 2 AMA guidelines are implemented by the pharmacotherapist. This more intensive intervention includes greater parental supervision of child diet and activity and goal setting for weight loss.

Investigators consult with a pediatric endocrinologist, and participants will be referred to a pediatrician if they cross any of the following thresholds and maintain them over two weeks:

• Substantial weight gain (i.e., ≥10% increase in baseline weight);

• diabetes mellitus (fasting glucose > 125 mg/dL on two occasions or GTT 120 min glucose ≥200 mg/dL);

• dyslipidemia with triglyceride or cholesterol levels > 450 mg/dL.

Children must be withdrawn from the study and be referred for individualized care in the community in any of 3 situations: 1) participant or guardian request, 2) judgment of the independent pediatrician or consulting pediatric endocrinologist that metabolic problems require treatment beyond that which the child is currently receiving in the study, or 3) significant worsening of psychiatric symptoms unless the PT and/or family request review by the study wide PI steering committee. Significant worsening of psychiatric symptoms is operationalized as IE rating of CGI-I of 6 – “much worse” or 7 – “very much worse” on two successive occasions over a period of at least two weeks.

There are no defined stopping criteria for weight gain or the development of metabolic problems. We decided to use the clinical consultation from an independent pediatric expert, rather than setting a predetermined stopping threshold because there are several considerations that need to be assessed to determine if a patient should seek alternative care. For example, families may not have access to alternative weight loss programs that can accommodate the child’s mental health needs (e.g. youth with developmental delay or aggressive behavior problems). Secondly, it may not be feasible (e.g. added appointment burden, travel time) for families to attend a weight loss program that is not integrated with the child’s mental health care. Finally, multiple clinical factors (e.g. family history, duration of metabolic abnormalities) should be evaluated to consider the child’s unique risk for diabetes and heart disease. Thus, we allowed for the decision about study discontinuation to be made clinically, rather than by a single strict metabolic stopping criterion.

## Conclusion

Child psychiatry clinical trials have traditionally focused on efficacy of medications to treat mental illness. IMPACT addresses a significant gap in current research by focusing on interventions to manage clinically relevant adverse effects. Adverse effect concerns are an important priority for research because of their short-term impact on treatment adherence and long term impact on a child’s development and risk for future health problems. SGA induced weight gain is a also major public health concern because SGA prescribing to youth is expected to increase with growing uses of these medications in pediatric treatment.

In addition to the focus on side effect concerns, there are several noteworthy aspects of the IMPACT study design. First, the study duration of six months is significantly longer than typical acute treatment studies. This longer time frame allows for more comprehensive assessment of psychiatric stability and is a more appropriate follow-up period for weight loss interventions, which often report that acute weight loss reverses over time. Secondly, the eligibility criteria include youth who are often excluded from traditional clinical trials, improving the generalizability of study results to real world practice. For example, youth with mild mental retardation, bipolar illness, co-occurring mental illness, and/or commonly co-prescribed pharmacologic agents have not been excluded. Thirdly, the state of the art metabolic assessment techniques, including body composition analysis and insulin sensitivity testing, allows for comprehensive and developmentally appropriate assessment of early cardiovascular disease and type 2 diabetes risk. With FDA approvals for pediatric antipsychotic treatment down to age 5–6 years old for some SGA medications, there is a growing need to identify metabolic changes that can be detected in young children (e.g. insulin sensitivity changes that proceed over diabetes).

One important challenge of the IMPACT study design has been the rapid changes in available medicines and prescribing trends. As described, increasing use of aripiprazole and discontinuation of molindone prompted design changes to the in-progress study. Moreover, three new antipsychotic medications have been made available in the US market since the IMPACT study has been initiated, namely asenapine, lurasidone, and iloperidone. Off label medication treatments in child psychiatry are common, and changes in clinical practice and standard of care sometimes precede pediatric studies to establish evidence base for treatments. It is unclear at this point if any of these new agents will have a more favorable risk to benefit ratio for the switch strategy than either aripiprazole or perphenazine. This rapidly changing psychopharmacology landscape raises important issues for study in terms of clinical treatment (e.g. factors that lead to rapid adoption of medication in pediatric treatment prior to established pediatric evidence base) as well as considerations for funding agencies in terms of the timeline and flexibility in conducting research so that the questions developed when the study is proposed remain relevant to real world prescribing practices when the results are available.

It is important to note that discontinuing antipsychotic treatment altogether is an option that may be considered clinically for youth with SGA associated weight gain based on patient/parent preference, severity of adverse events, and current psychiatric stability/symptom profile. Since most youth prescribed antipsychotic medication are being treated for non-psychotic conditions, it is appropriate to exhaust first line medication and psychosocial treatments before even initiating SGA medication and to monitor for changes in the risk to benefit ratio of SGA medication over the course of treatment. In our sample, most referred youth had a history of significant safety concerns (e.g. severe aggression, self-harm), high-level service utilization (e.g. inpatient, emergency room services), and recurrent episodes of mental illness prior to SGA treatment. Thus, the risk to benefit ratio of treatment options needs to take into consideration both metabolic and psychiatric health concerns.

In summary, results from the IMPACT study are expected to provide important and currently lacking information that can help guide clinical decision making aimed at improving both psychiatric and physical health of youth with severe mental disorders.

## Competing interests

The authors declare that they have no competing interests.

## Authors’ contributions

MR, CC, LS, and GR are the PI’s of the study, RH and JJ are the biostatistician experts, and CK is the lead study coordinator. All authors participated in study-wide steering committee conference calls to develop the outline of the paper and discuss feedback on manuscript drafts. GR and CK developed the initial draft, with sections developed by MR and CC on the study background/rationale; RH and JJ on the data analysis plan; and LS on design challenges. RH, JJ and AS from the study-wide data center provided significant input on sections that addressed DSMB/safety monitoring protocols. All remaining authors conducted research assessments of participants in their role as pharmacotherapists, independent evaluators, and research assistants/study coordinators (including healthy lifestyle education teaching). All authors have also participated in weekly to bi-weekly steering committee calls to discuss ongoing study design issues. All authors have read and approved the manuscript for publication.

## Disclosures

The aripiprazole for this study was donated by Bristol-Myers Squibb. Dr. Sikich has received funding from Bristol-Meyers Squibb for a sponsored clinical trial and donation of medication and patient travel expenses for an investigator initiated study of aripiprazole. Dr. Correll has been a consultant, advisor, lecturer and/or data safety monitor to or has received honoraria from: Actelion, Alexza; AstraZeneca, Biotis, Boehringer-Ingelheim, Bristol-Myers Squibb, Cephalon, Desitin, Eli Lilly, Genentech, Gerson Lehrman Group, GSK, IntraCellular Therapies, Lundbeck, Medavante, Medicure, Medscape, Merck, National Institute of Mental Health, Novartis, Ortho-McNeill/Janssen/J&J, Otsuka, Pfizer, ProPhase, Roche, Schering-Plough, Sepracor/Sunovion, Supernus, Takeda, Teva and Vanda. He has received grant support from BMS, Feinstein Institute for Medical Research, Janssen/J&J, National Institute of Mental Health (NIMH), National Alliance for Research in Schizophrenia and Depression (NARSAD), and Otsuka. Dr. Hamer has been a consultant, advisor, or data safety monitor to or has received honoraria from: Abbott, Acadia, Allergan, Alkermex, Alpharma, AstraZeneca, Cenerex, Corcept, Eli Lilly, Endo, Epix, J&J, NeuroPharmaBoost, Novartis, PureTech ventures, Pfizer, Roche, Sanofi-aventis, Schwartz, Solvey, Takeda, Wyeth, and NeurogensX, Inc. Dr. Hamer and/or his spouse own stock in Bristol-Myers Squibb, Amgen, Lilly, genetech, Proctor and Gamble, and Sepracor.
